# Identification and Therapeutic Outcome Prediction of Cervical Spondylotic Myelopathy Based on the Functional Connectivity From Resting-State Functional MRI Data: A Preliminary Machine Learning Study

**DOI:** 10.3389/fneur.2021.711880

**Published:** 2021-10-08

**Authors:** Qian Su, Rui Zhao, ShuoWen Wang, HaoYang Tu, Xing Guo, Fan Yang

**Affiliations:** ^1^Tianjin Key Laboratory of Cancer Prevention and Therapy, Department of Molecular Imaging and Nuclear Medicine, Tianjin Medical University Cancer Institute and Hospital, National Clinical Research Center for Cancer, Tianjin's Clinical Research Center for China, Tianjin, China; ^2^Department of Orthopedics Surgery, Tianjin Medical University General Hospital, Tianjin, China; ^3^School of Basic Medical Sciences, Tianjin Medical University, Tianjin, China; ^4^School and Hospital of Stomatology, Tianjin Medical University, Tianjin, China; ^5^Department of Radiology, Tianjin Medical University General Hospital, Tianjin, China

**Keywords:** rs-fMRI, machine learning, cervical spondylotic myelopathy, support vector machine, functional connectivity

## Abstract

Currently, strategies to diagnose patients and predict neurological recovery in cervical spondylotic myelopathy (CSM) using MR images of the cervical spine are urgently required. In light of this, this study aimed at exploring potential preoperative brain biomarkers that can be used to diagnose and predict neurological recovery in CSM patients using functional connectivity (FC) analysis of a resting-state functional MRI (rs-fMRI) data. Two independent datasets, including total of 53 patients with CSM and 47 age- and sex-matched healthy controls (HCs), underwent the preoperative rs-fMRI procedure. The FC was calculated from the automated anatomical labeling (AAL) template and used as features for machine learning analysis. After that, three analyses were used, namely, the classification of CSM patients from healthy adults using the support vector machine (SVM) within and across datasets, the prediction of preoperative neurological function in CSM patients *via* support vector regression (SVR) within and across datasets, and the prediction of neurological recovery in CSM patients *via* SVR within and across datasets. The results showed that CSM patients could be successfully identified from HCs with high classification accuracies (84.2% for dataset 1, 95.2% for dataset 2, and 73.0% for cross-site validation). Furthermore, the rs-FC combined with SVR could successfully predict the neurological recovery in CSM patients. Additionally, our results from cross-site validation analyses exhibited good reproducibility and generalization across the two datasets. Therefore, our findings provide preliminary evidence toward the development of novel strategies to predict neurological recovery in CSM patients using rs-fMRI and machine learning technique.

## Introduction

Cervical spondylotic myelopathy (CSM) is the most common cause of non-traumatic spinal cord injury (1–3). As a non-invasive and effective approach for evaluating structural damage of CSM, several neuroimaging techniques targeting the cervical spine to diagnose and to predict neurological recovery in CSM have been investigated. Currently, cervical structural MRI is regarded as a gold standard for diagnostic and prognostic imaging for CSM in clinical practice (4, 5). However, there are insufficient empirical data, due to limited anatomical information from the cord structure, to support the usage of conventional structural cervical MRI (e.g., T1-weighted and T2-weighted images) as a predictive biomarker of postoperative neurological recovery (6). Therefore, the need for simple, accurate, and non-invasive imaging biomarkers for diagnosing and predicting neurological function recovery in CSM patients is warranted (5).

As a non-invasive imaging technique measuring the functional changes in CSM, the brain resting-state functional MRI (rs-fMRI) has been proved to successfully identify the CSM patients from healthy participants (7–13). In contrast to conventional MRI technique, which only measures the structural damages within the conduction pathway, rs-fMRI measures the brain activities that encompass information for all motor and cognitive functions as the brain functions as a “control and data center.” Therefore, CSM-associated information is distributed in widespread regions of the brain (8, 11, 14). Therefore, several studies conducted the rs-fMRI to predict the neurological recovery of CSM patients following decompression surgery. Takenaka et al. found that the functional connectivity (FC) between certain brain regions associated with postoperative gain in the 10-s test might be sufficient to provide a prediction formula for potential recovery (11). Moreover, they also found that the resting-state amplitude of low-frequency fluctuation is also a potentially prognostic functional biomarker in cervical myelopathy (15).

Their studies provided new insights for developing a novel method for diagnostic and prognostic imaging in CSM patients. However, a major limitation is that their results were mainly using mass univariate analyses (e.g., correlation analysis and linear regression), which can simply measure the association between average regional activity amplitude and clinical measures. Given that the rs-fMRI data consist of massive variables measuring the functional state of the brain and the interrelationship between these variables, the univariate analyses thus may miss the information associating with the CSM pathology. Rapid advancement of multivariate pattern analysis (MVPA) of fMRI data (16, 17) offers the unprecedented ability to detect small differences in spatial patterns of functional brain changes and reorganizations between disease-state and disease-free conditions (18, 19). Also, MVPA approaches evaluate the complexity interaction among massive variables, hence making accurate predictions (16, 20–22). The support vector machine (SVM) has been regarded as one of the MVPA techniques showing high accuracy in diagnosing and predicting clinical measures in various diseases using fMRI data (20, 23). The SVM is a supervised-learning model that analyzes data used for classification and regression analysis. The SVM technique has a great potential in defining a set of features from various regions of the brain, allowing the classification of healthy controls (HCs) and patients, and yields a potential translational impact (16, 24).

Therefore, to establish a model with potential diagnostic and prediction properties of clinical outcomes in patients with CSM, we aimed to test the utility of FC, which integrates spatial relationships among different brain regions and is the most widely used metric among other analytical methods in rs-fMRI studies (9, 25–27), as a potential biomarker for diagnosing and predicting surgical outcomes in CSM patients using the SVM approach. Moreover, FC has been shown to be one of the most reliable metrics (i.e., cross-scan stability) in fMRI studies (28–30). In this study, we performed an MVPA to classify CSM patients and HCs, both with and without feature selection *via* SVM. We then used support vector regression (SVR) to predict the preoperative Japanese Orthopedic Association (JOA) scores, JOA recovery rate, and the JOA recovery scores following spinal cord decompression surgery. We also tested the reproducibility and generalizability of our results by cross-validation between the two independent datasets. To the best of our knowledge, this is the first study testing the utility of combining rs-FC and machine learning method for diagnosing and predicting surgical outcomes in CSM patients.

## Materials and Methods

### Study Subjects

The local Institutional Review Board of Tianjin Medical University General Hospital (Tianjin, China) approved this cross-sectional, retrospective study. Written informed consent was obtained from all participants before each procedure during the data collection.

In this study, two datasets (i.e., two pre-established databases) obtained in Tianjin Medical University General Hospital at two different time frames were included: the first dataset involved 27 right-handed CSM patients pooled from 2015 to 2016. The inclusion criteria of CSM patients into this dataset (dataset 1) included the following: (1) meet a criterion for diagnosing the CSM (i.e., clear evidence of cord compression on cervical spine MRI, explicit clinical manifestations of sensorimotor extremities' deficits or bladder, and bowel dysfunction); (2) no clinical evidence or history of any other diseases including neurological diseases, psychiatric diseases, ocular diseases, systematic diseases, brain diseases, extracranial vertebral artery, and carotid artery; (3) no history of alcohol and substance abuse; (4) the patients agreed to undergo decompression of spinal canal, had no previous history of cervical spinal surgery, and are able to complete the functional MRI studies. Furthermore, 11 healthy subjects with similar age, gender, and academic years (i.e., with differences for age, academic years all below 2 years from a given subject in the patient group) were recruited through advertisements. Only the healthy subjects with no evidence of spinal compression, no ocular disease, no other spinal or brain neurological disorders or systemic disease, and able to complete the fMRI studies were included—details of study participants were as per our previous study (31). In the second dataset (dataset 2), 26 CSM patients and 36 HCs sampled from 2019 to 2020 were recruited in our study using the inclusion criteria for the first dataset; details of study participants were as per our previous study (32). Therefore, a total of 53 CSM patients and 47 healthy participants were included in our current study.

The detailed order for data collection were as follows: (1) the patients were first examined and evaluated by a senior orthopedic surgeon 1 week before surgery for acquiring preoperative JOA scores; subsequently, the patients underwent fMRI scan for acquiring preoperative fMRI data; (2) the patients underwent spinal cord decompression surgery; and (3) all patients were reevaluated by the same surgeon at the clinics 6 months after surgery to acquire the postoperative JOA scores.

### Acquisition of MRI Data and Preprocessing

For dataset 1, data were acquired using a 3.0T magnetic resonance scanner (Discovery MR750; General Electric Healthcare, Chicago, IL, USA) with an eight-channel phased-array head coil. Before scanning, earplugs were placed inside the subjects' ears to keep out noise. The subjects were then instructed to fix their heads with sponge pads to minimize unconscious activity. Subjects could keep their eyes closed but remain awake and avoid specific and strong ideological activities during scanning. We made clear instruction to the participants that they should not fall asleep during the entire scan. We also confirmed with the participants that they have been awake during the entire scan after they completed the scan. Afterward, functional images of the brain were captured using a gradient echo-planar imaging (EPI) sequence at the following parameters: repetition time (TR) = 2,000 ms; echo time (TE) = 30 ms; flip angle (FA) = 90°; field of view (FOV) = 240 mm × 240 mm; matrix = 64 × 64; the number of slices = 38 slices; and slice thickness = 3.0 mm. A total of 180 images were obtained within 6 min. Structural images were captured using a three-dimensional T1-weighted image (3D T1WI) for co-registration and normalization of functional images. The parameters of the 3D T1WI were as follows: sagittal acquisition; TR = 7.8 ms; TE = 3.0 ms; inversion time = 450 ms; FA = 13°; FOV = 256 mm × 256 mm; matrix, 256 × 256; number of slices = 180; and slice thickness = 1.0 mm.

For the second dataset (dataset 2), the 3T fMRI data were acquired using a MAGNETOM Prisma 3T MR scanner (Siemens, Erlangen, Germany) with a 64-channel phase-array head-neck coil. Preparation of the study subjects was identical to that described in dataset 1. Blood oxygenation level-dependent (BOLD) signals were detected with a simultaneous multi-slice gradient EPI sequence at the following parameters: TE = 30 ms; TR = 800 ms; FOV = 222 mm × 222 mm; matrix = 74 × 74; in-plane resolution = 3 mm × 3 mm; FA = 54°; slice thickness = 3 mm; gap = 0 mm; number of slices = 48; slice orientation = transversal; bandwidth = 1,690 Hz/pixel; parallel acquisition technique (PAT) mode; slice acceleration factor = 4; and phase-encoding acceleration factor = 2. A total of 450 images were captured in a period of 6 min. A high-resolution 3D T1 structural image [two inversion contrast magnetization-prepared rapid gradient echo (MP2RAGE)] was also acquired at the following parameters: TR/TE = 4,000 ms/3.41 ms; inversion times (TI1/TI2) = 700 ms/2,110 ms; FA1/FA2 = 4°/5°; matrix = 256 × 240; FOV = 256 mm × 240 mm; number of slices = 192; in-plane resolution = 1 mm × 1 mm; slice thickness = 1 mm; slice orientation = sagittal; and total duration = 6 min 42 s.

All MRI data were preprocessed using the toolbox Data Processing Assistant for rs-fMRI (DPARSF; http://www.restfmri.net/forum/DPARSF) procedure from which 180 volumes were acquired for functional scan in dataset 1 and 450 volumes in dataset 2. The first 10 volumes from each functional scan were excluded from the subjects to correct acclimatization to the scanning environment and magnetization stabilization. A slice-timing correction was performed (not done in dataset 2 since the TR of dataset 2 was significantly shortened); and motion correction was performed to remove timing differences and head movement. The functional images were co-registered with the structural images and spatially normalized to the Montreal Neurological Institute template, where each voxel was resampled to 3 × 3 × 3 mm^3^. Subsequently, the resampled images were smoothed with an 8-mm full-width-at-half-maximum isotropic Gaussian kernel. After that, the linear trend and bandpass filter (0.01~ 0.08 Hz) were applied to remove the effects of high-frequency noise. Finally, six motion parameters, the mean global signal, the white matter signal, and the cerebrospinal fluid (CSF) signal were extracted as covariates to reduce the non-neural signal. The resulting data were subjected to further analysis.

### Clinical Assessment

A group of senior spine surgeons performed clinical assessments including JOA evaluation (33). The clinical diagnosis of CSM was based on the neurological signs and symptoms in patients together with relevant radiological findings of stenosis. The JOA was used preoperatively and postoperatively after 6 months for clinical evaluation. The JOA recovery scores were calculated for the study group by subtracting preoperative JOA scores from postoperative JOA scores.

The JOA recovery rate was defined as follows:


$$\begin{array}{l} JOA\;recovery\;rate=\\ \;\frac{\left(Postoperative\;JOA\;scores-Preoperative\;JOA\;scores\right)}{\left(17-Preoperative\;JOA\;scores\right)} \end{array}$$

And the JOA recovery was defined as follows:


$$\begin{array}{l} JOA\;recovery=Postoperative\;JOA\;scores\\ \;-Preoperative\;JOA\;scores \end{array}$$

### Functional Connectivity Analysis

**Figure 1:**
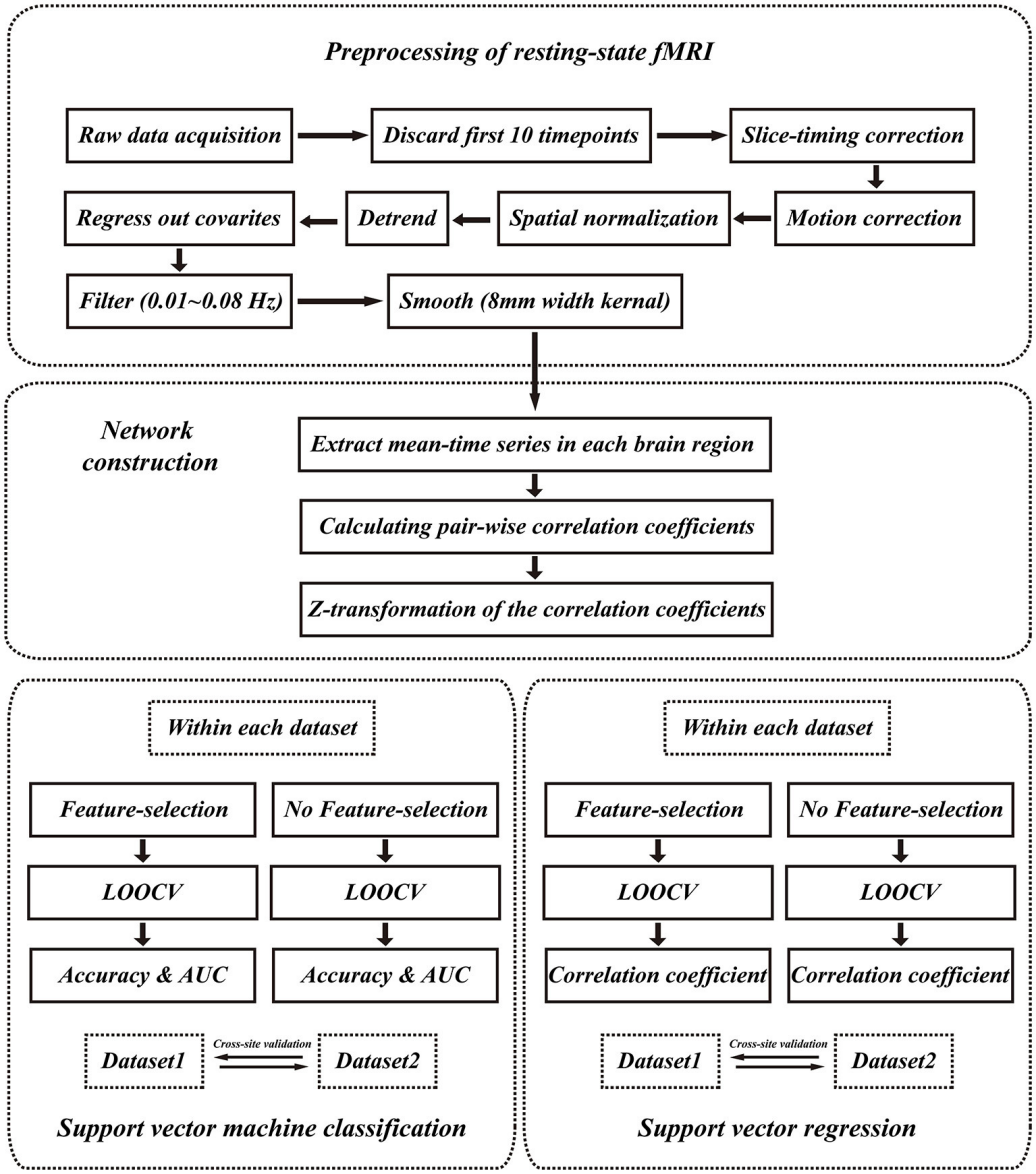
Analysis pipeline. The analysis pipeline of our study. LOOCV, leave-one-out cross-validation.

A total of 116 functionally defined regions of interest (ROIs) were selected using an automated anatomical labeling (AAL) template (34). The average resting-state BOLD time series for each ROI were then extracted and then correlated with the BOLD time series of every other ROI using Pearson's correlation for every subject. From the resulting square (116 × 116) symmetric matrix of correlation coefficients for each subject, only 6,670 ROI-pair correlation values from the lower triangular part of the matrix were retained, and the redundant elements from the upper triangular part of the matrix (i.e., the upper triangular part is identical to the lower triangular part), and diagonal elements were excluded. The 6,670 ROI pairs were subjected to Fisher's z-transformation for normalization and used as features for further analyses. Figure 1 shows a series of steps in a representative pipeline of the classification method used in this study.

### Mass Univariate Analyses

Mass univariate analyses were performed to reveal the FC differences between CSM patients and HCs. The two-sample *t*-test was performed for each FC (i.e., FCs calculated between each pair of brain regions) using age, gender, scan parameters, and education as covariates. Therefore, 6,670 *p*-values were obtained. Subsequently, all *p*-values were corrected for multiple comparisons with false discovery rate (FDR), corresponding to a corrected *q* < 0.05. This analysis was also repeated within each dataset to give a detailed result of each dataset.

### Classification of Cervical Spondylotic Myelopathy From Healthy Adults

The pattern classification was performed to classify patients with CSM and HCs based on FC using the MVPANI toolbox (http://funi.tmu.edu.cn) and LibSVM's implementation of linear SVM using the default parameters (35). A large vector with 6,670 features was extracted from each subject.

For within-dataset analyses, the leave-one-out-validation (LOOCV) technique was employed to overcome the loss of generalization due to the small training and testing sample size in this study. The bias of LOOCV error was expected to be small since almost the entire dataset was used for training, and the trained model was close to the real one.

In LOOCV, for example (e.g., within dataset 1), (1) one data point in dataset 1 was held out (i.e., treated as the testing sample), and the model was trained vis-à-vis the rest of the data within this dataset and then tested with that held-out data point (i.e., one fold). Subsequently, the classification accuracy for the testing sample was obtained (i.e., the classification accuracy of this fold). (2) This procedure was repeated until all data points were held out once as the testing sample, (3) The average classification accuracy across all folds was obtained for this dataset. A feature selection procedure embedded within the LOOCV procedure was also performed. For each fold in LOOCV, all features were initially used to train a classifier using the training dataset and then ranked from high to low according to the resultant feature weights (e.g., absolute value). Then, the top 5% of the features with the highest weights were selected and used to train a new classifier using the training dataset. Afterward, the obtained classifier was tested using the test dataset, resulting in classification accuracy for this LOOCV step. Therefore, classification accuracy was obtained for every LOOCV step, and then the absolute accuracy was calculated as the average across all LOOCV steps. The feature selection procedure was repeated for a series of selected features from 5 to 100% with a step of 5% increment, resulting in 20 selected feature sets with 20 averaged classification accuracies. For each of the 20 classification accuracies, the corresponding *p*-value was calculated from the null distribution obtained from 1,000 random permutation tests by randomly shuffling the labels of subjects in the training dataset, with the selected corresponding feature set in each LOOCV step. The *p*-values were calculated as a proportion of the number of permutations generated that were greater than or equal to actual classification accuracy, and the total number of permutations. If none of the 1,000 permutations reached the actual accuracy, the *p*-value was labeled as *p* < 0.001. Note that in this procedure, 20 independent MVPAs were analyzed with a different percentage in feature selection. Thus, the *p*-values that were calculated from the permutation tests were further corrected for multiple comparisons using the Bonferroni correction method, where *p* < 0.05/20 = 0.0025 was considered statistically significant. All LOOCV and feature-selection steps were also performed within dataset 2.

Generalization of the SVM model was evaluated by a cross-site validation test between two datasets, where each dataset was treated as a testing set once, and not involved in the training process. The brief description of the cross-site validation test was as follows: (1) the SVM model was trained using the data of dataset 1 and then tested on the data of dataset 2. (2) Subsequently, the classification accuracy was obtained for this validation step. (3) The SVM model was trained using the data of dataset 2 and then tested on the data of dataset 1. (4) The classification accuracy was also obtained for this validation step. (5) The mean classification accuracy of all validation steps (i.e., two accuracies) were obtained for cross-site validation analysis. In addition to classification accuracy, the receiver operating characteristic (ROC) curves and the corresponding area the under curve (AUC) for within-dataset and cross-dataset classification were also calculated.

### Prediction of Preoperative Japanese Orthopedic Association Scores, Japanese Orthopedic Association Recovery Rate, and Japanese Orthopedic Association Recovery Scores in Cervical Spondylotic Myelopathy

The presurgical FC of each subject, as the training feature, was used to establish and evaluate SVR models. The SVR models were used to predict the preoperative JOA scores, JOA recovery rate, and JOA recovery scores. The SVR analyses embedded with LOOCV were also performed within each dataset and across datasets.

In within-dataset analyses, LOOCV procedure was also performed (e.g., within dataset 1): (1) one data point in dataset 1 was held out, and the model was trained vis-à-vis the rest data within the dataset; (2) then the trained model was tested with that held-out data point. For this procedure, a predicted value was obtained, representing a predicted value for this subject (i.e., held-out data point). This procedure was repeated until all data points were held out once. A feature selection procedure that was embedded within the LOOCV procedure was performed. The detailed procedure was similar to the description in *Classification of Cervical Spondylotic Myelopathy From Healthy Adults* in the *Materials and Methods*. In this section, the correlation coefficients between the predicted labels and actual labels were calculated and used for deriving the corresponding *p*-values from null distribution. The detailed information of the LOOCV and feature-selection procedures was as follows: for each LOOCV step, all features were initially correlated with the actual label, and the corresponding R and *p*-values were obtained. Features with a *p*-value of < 0.05 were selected and used to train a regression model with the training dataset. The regression model was tested using the test dataset, thereby yielding the predicted labels for the test data.

Evaluation of the generalizability of the SVM model was performed using a cross-site validation test between two datasets where each dataset that was not involved in the training process was held as testing set once.

The brief description of the cross-site validation is as follows: (1) the SVR model was trained using the data of dataset 1 and then tested on the data of dataset 2. (2) Subsequently, the predicted labels of each data point in dataset 2 (i.e., testing sample) were obtained for calculating the correlation coefficients and root mean square error (RMSE) (e.g., between predicted labels and actual labels; dataset 1 as the training data). (3) The SVM model was trained using the data of dataset 2 and then tested on the data of dataset 1. (4) Subsequently, the predicted labels of each data point in dataset 1 (i.e., testing sample) were obtained for calculating the correlation coefficients and RMSE (e.g., between predicted labels and actual labels; dataset 2 as the training set). The corresponding *p*-value was derived from the null distribution that was obtained from 1,000 random permutation tests, by randomly shuffling the labels of the subjects in the training dataset, with the corresponding feature set. Specifically, the *p*-values were determined as a proportion of the number of permutations greater than or equal to the actual correlation coefficient (and the proportion of the number of permutations smaller than or equal to the RMSE) and the total permutations. If none of the 1,000 permutations reached the actual correlation coefficient (or smaller than the actual RMSE), the *p*-value was considered to be *p* < 0.001. Pearson's correlation analysis can only provide the linear association between the predicted labels and actual labels, while the Bland–Altman analysis could further describe the agreement between two variables (i.e., predicted label and actual label) and help to determine the true limits of agreement (LOA) for each prediction procedure. Therefore, the Bland–Altman analyses would significantly aid interpretation of the clinical impact of these analyses.

## Results

### Clinical Measures and Demographic Data

The preoperative, postoperative, and recovery JOA scores are presented in Table 1. No significant differences in age, gender, and academic years were observed between CSM patients and HCs.

**Table 1:** Demographic data and clinical assessment.

**Dataset 1**				**Dataset 2**			
**Characteristic**	**CSM (*n* = 27)**	**HC (*n* = 11)**	***p*-value**	**Characteristic**	**CSM (*n* = 26)**	**HC (*n* = 36)**	***p*-value**
Age (years)	57.9 ± 9.1	54.8 ± 8.4	0.34	Age (years)	54.7 ± 8.8	53.7 ± 8.3	0.54
Gender (female/male)	12/15	5/6	0.96	Gender (female/male)	12/14	17/19	0.93
Education (years)	10.8 ± 2.7	11.6 ± 2.5	0.42	Education (years)	10.7 ± 2.5	11.2 ± 23	0.41
Pre-JOA	11.8 ± 1.5			Pre-JOA	11.0 ± 1.8		
Post-JOA	15.7 ± 2.3			Post-JOA	14.2 ± 2.6		
JOA recovery	3.9 ± 1.8			JOA recovery	3.1 ± 2.4		

*JOA, Japanese Orthopedic Association score; Pre, preoperative; Post, postoperative; CSM, cervical spondylotic myelopathy; HC, healthy control.*

*JOA recovery = Postoperative JOA scores – Preoperative JOA scores*.

### Mass Univariate Analyses

**Figure 2:**
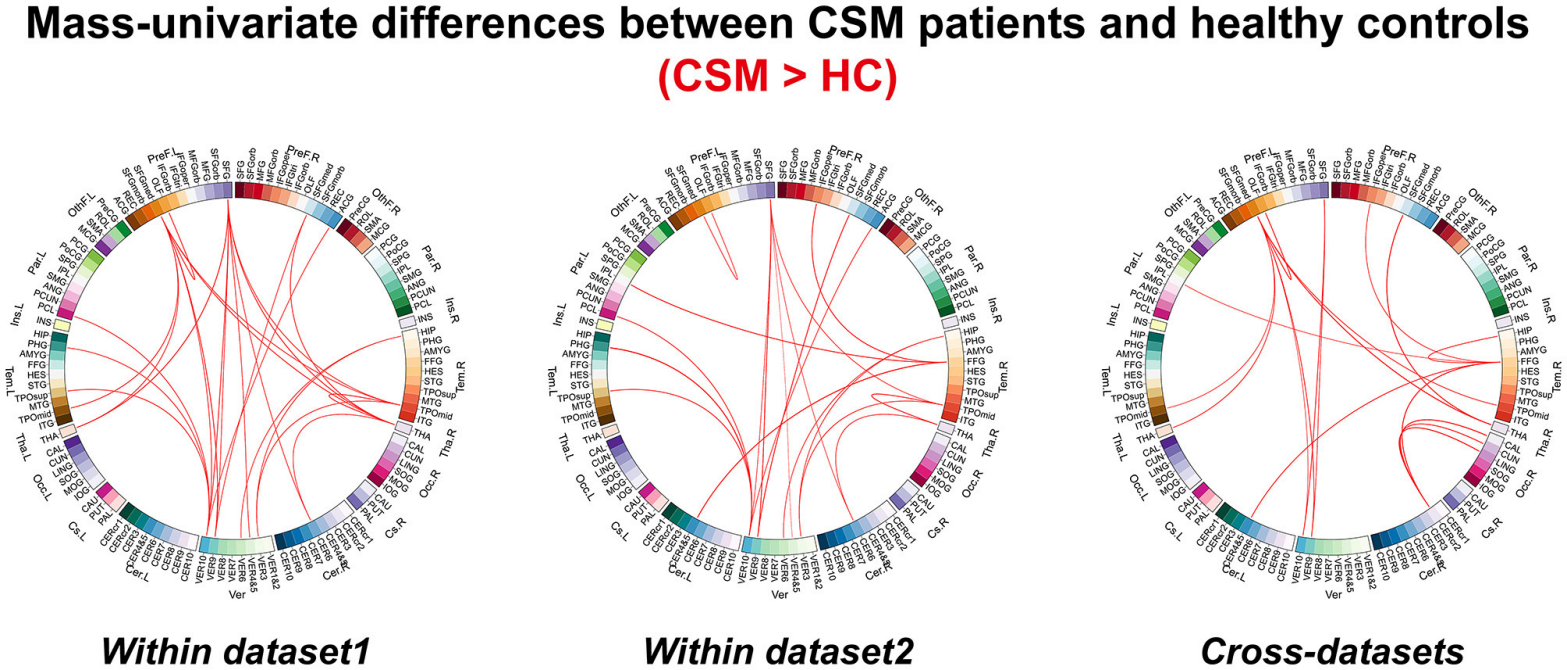
The differences for functional connectivity between cervical spondylotic myelopathy patients and healthy controls revealed by mass univariate analyses.

The FC differences between CSM patients and HCs are shown in Figure 2. Increased FCs (i.e., FCs were increased in CSM in comparison with HC participants) were obtained both within dataset and across dataset analyses. In dataset 1, increased FCs are mainly between the frontal lobe and cerebellum, frontal lobe and thalamus, and temporal lobe and thalamus. In dataset 2, increased FCs are mainly between the frontal lobe and cerebellum, and temporal lobe and cerebellum.

### Classification of Cervical Spondylotic Myelopathy From Healthy Adults

**Figure 3:**
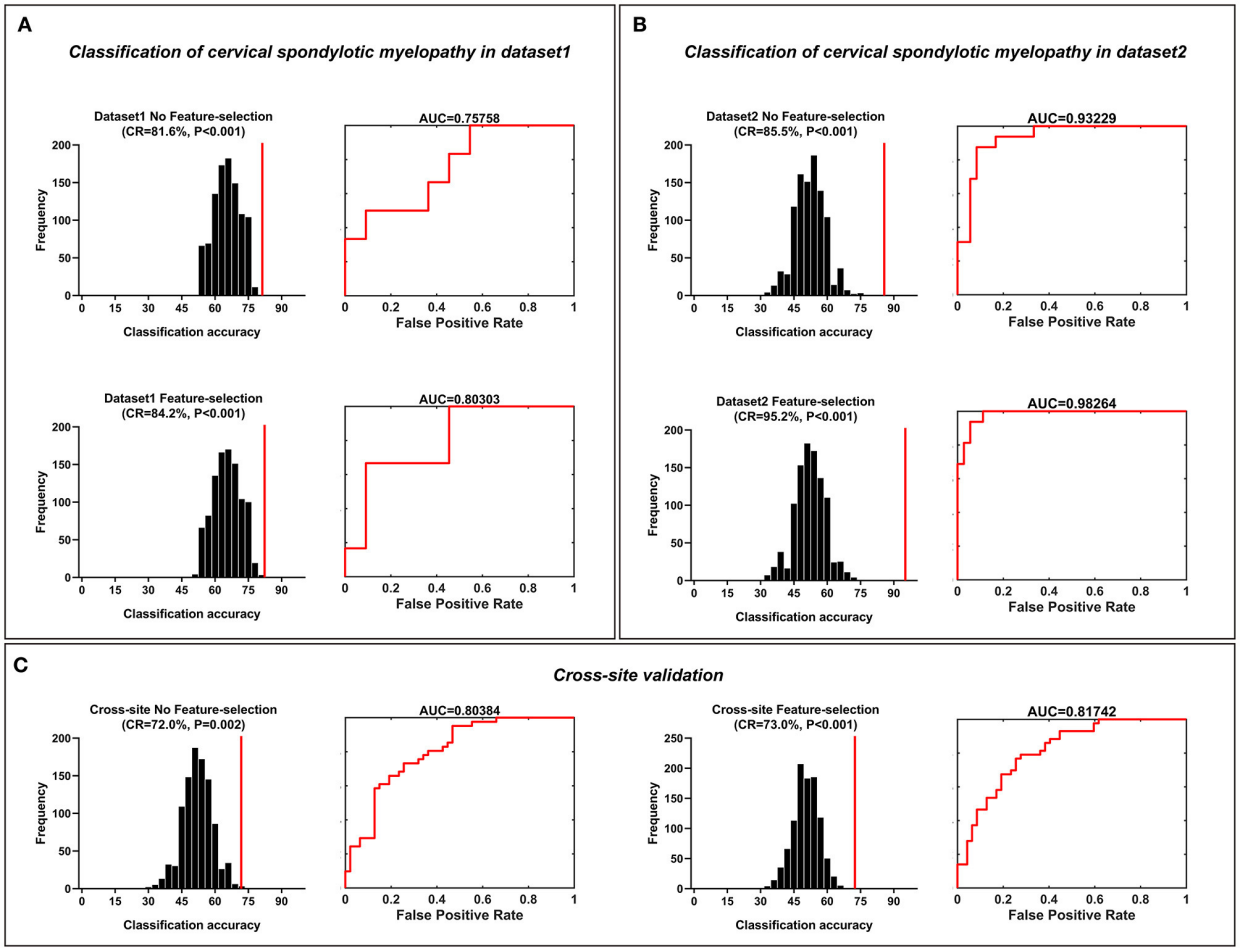
Classification of cervical spondylotic myelopathy patients from healthy controls. Classification of cervical spondylotic myelopathy from healthy adults. **(A)** The classification accuracies obtained from both feature-selection and no-feature-selection models in dataset 1. **(B)** The classification accuracies obtained from both the feature-selection model and the no-feature-selection model in dataset 2. **(C)** The classification accuracies obtained both from both the feature-selection and no-feature-selection models during the cross-site validation procedure. The corresponding ROC curve and AUC were also illustrated. CR, correct rate; ROC, receiver operating characteristic; AUC, area the under curve.

The SVM results are shown in Figure 3. The classification accuracies that were obtained from a no-feature selection procedure for each dataset and cross-site validation were 81.6% (*p* < 0.001, with Bonferroni correction) for dataset 1, 85.5% (*p* < 0.001) for dataset 2, and 72.0% (*p* = 0.002, with Bonferroni correction) for cross-site validation. The corresponding AUCs of ROC curves were 0.76 for dataset 1, 0.93 for dataset 2, and 0.80 for cross-site validation. The highest classification accuracies that were obtained with a feature selection procedure for each dataset and cross-site validation were 84.2% (*p* < 0.001, with Bonferroni correction) for dataset 1 (the model trained with top 25% features, 1,668 FC pairs), 95.2% (*p* < 0.001, with Bonferroni correction) for dataset 2 (the model trained with top 30% features, 2,001 FC pairs), and 73.0% (*p* < 0.001, with Bonferroni correction) for cross-site validation (the model trained with top 15% features, 1,001 FC pairs). The corresponding AUCs of ROC curves were 0.80 for dataset 1, 0.98 for dataset 2, and 0.82 for cross-site validation.

### Prediction of Preoperative Japanese Orthopedic Association Scores, Japanese Orthopedic Association Recovery Rate, and Japanese Orthopedic Association Recovery Scores in Cervical Spondylotic Myelopathy

**Figure 4:**
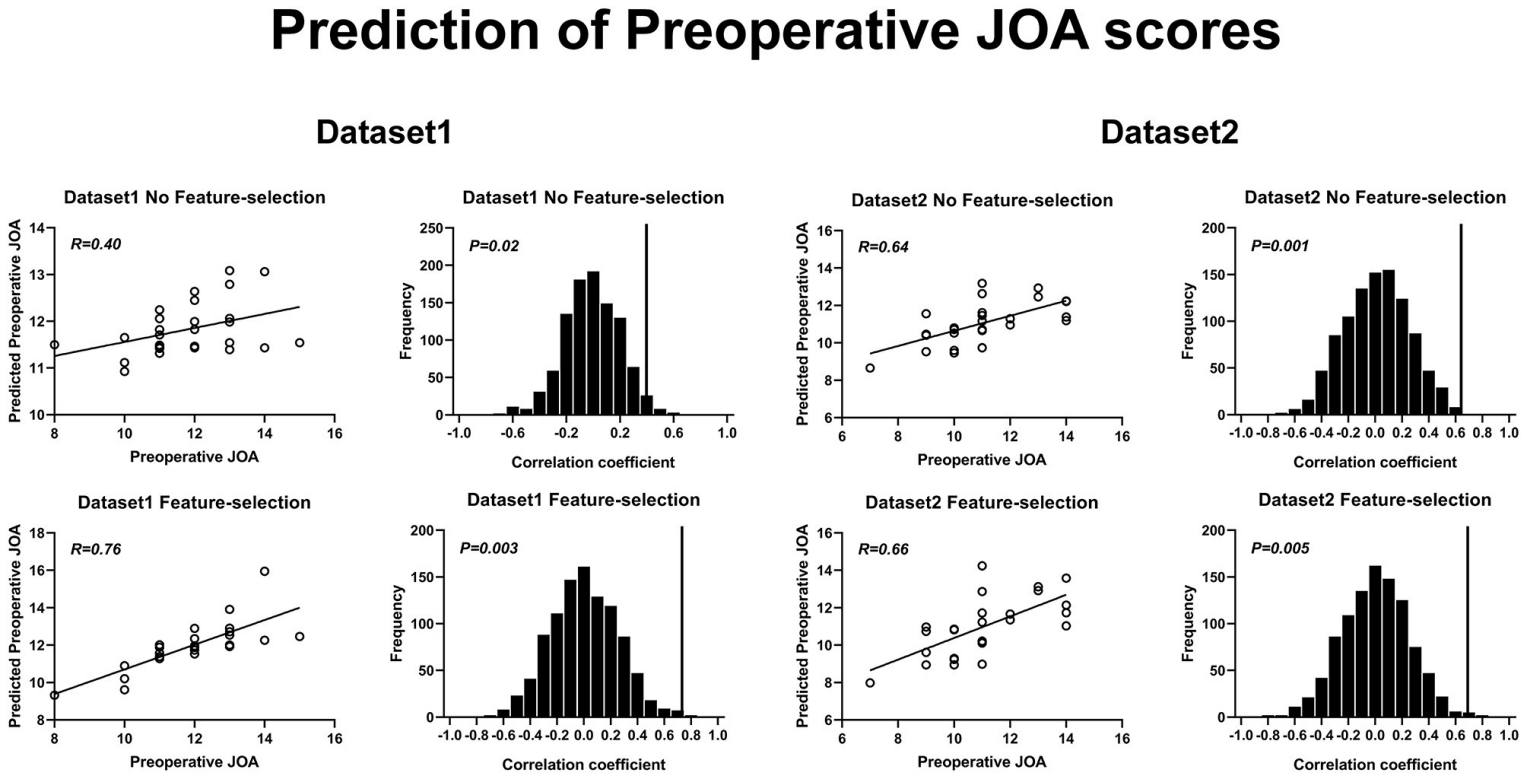
Prediction of preoperative JOA scores using rs-FC. Prediction of preoperative JOA scores. JOA, Japanese Orthopedic Association; rs-FC, resting-state functional connectivity.

The SVR results of preoperative JOA score predictions are shown in Figure 4. The correlation coefficients obtained with a no-feature selection procedure, between the predicted preoperative JOA scores and the actual preoperative JOA scores, were 0.40 (*p* = 0.02) for dataset 1 and 0.64 (*p* = 0.001) for dataset 2. The RMSE obtained with a no-feature selection procedure, between the predicted preoperative JOA scores and the actual preoperative JOA scores, were 0.259 (*p* < 0.05, Table 2) for dataset 1 and 0.262 (*p* < 0.005, Table 2) for dataset 2.

**Table 2:** The root mean square error (RMSE) for prediction analyses.

	**Pre-JOA**		**JOA recovery**		**JOA recovery rate**	
	**Feature selection**	**No feature selection**	**Feature selection**	**No feature selection**	**Feature selection**	**No feature selection**
	Within site					
Dataset 1	0.179^**^	0.259^*^	0.131^**^	0.193^*^	1.549^**^	1.831^*^
Dataset 2	0.257^**^	0.262^**^	0.365^*^	0.349^*^	4.342^**^	4.752^*^
	Cross-site validation					
Dataset 1 as training set	0.326^***^	0.347	0.260^**^	0.363	3.560^***^	6.736
Dataset 2 as training set	0.236^**^	0.326	0.258^*^	0.263	4.475^*^	5.860

*The root mean square error for the within-site and cross-site predictions of preoperative JOA scores, JOA recovery, and JOA recovery rate.*

* *p < 0.05,*

** *p < 0.005,*

*** *p < 0.001. Correlation coefficients with significant P values (P < 0.05) were shown in bold format*.

The correlation coefficients obtained with a feature selection procedure, between the predicted preoperative JOA scores and the actual preoperative JOA scores, were 0.76 (*p* = 0.003) for dataset 1 (46 FC pairs) and 0.66 (*p* = 0.005) for dataset 2 (22 FC pairs). The RMSE obtained with a feature selection procedure, between the predicted preoperative JOA scores and the actual preoperative JOA scores, was 0.179 (*p* < 0.005, Table 2) for dataset 1 and 0.257 (*p* < 0.005, Table 2) for dataset 2.

**Figure 5:**
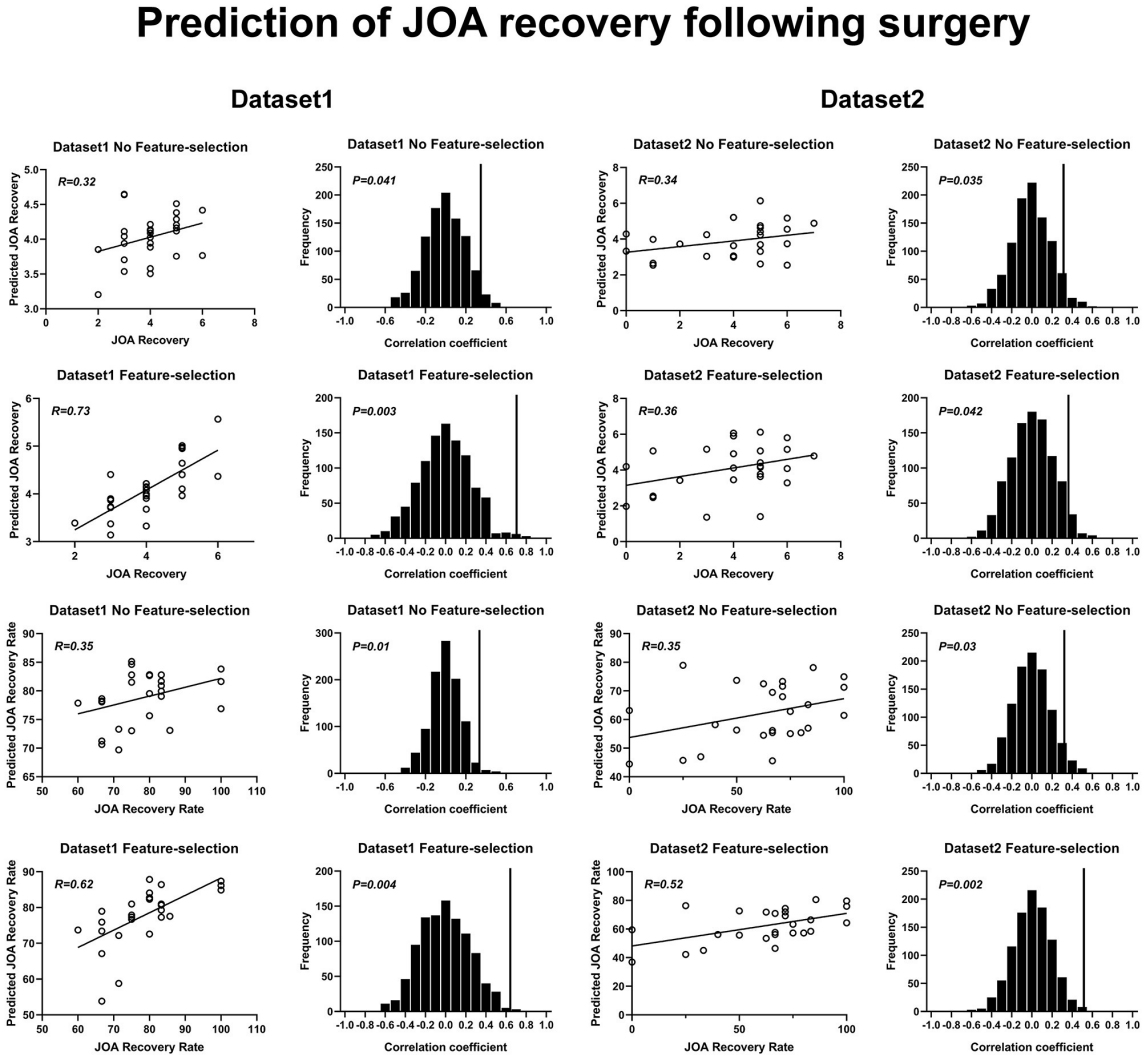
Prediction of JOA recovery using rs-FC. Prediction of preoperative JOA scores. JOA, Japanese Orthopedic Association; rs-FC, resting-state functional connectivity. JOA recovery = postoperative JOA scores minus preoperative JOA scores.

The SVR results of JOA recovery prediction are shown in Figure 5. The correlation coefficients obtained with a no-feature selection procedure, between the predicted JOA recovery scores and the actual JOA recovery scores, were 0.32 (*p* = 0.04) for dataset 1 and 0.34 (*p* = 0.035) for dataset 2. The correlation coefficients obtained with a feature selection procedure, between the predicted preoperative JOA scores and the actual preoperative JOA scores, were 0.73 (*p* = 0.003) for dataset 1 (51 FC pairs) and 0.36 (*p* = 0.04) for dataset 2 (18 FC pairs). The RMSE obtained with a no-feature selection procedure, between the predicted JOA recovery scores and the actual JOA recovery scores, was 0.193 (*p* < 0.05, Table 2) for dataset 1 and 0.349 (*p* < 0.05, Table 2) for dataset 2. The RMSE obtained with a feature selection procedure, between the predicted JOA recovery scores and the actual JOA recovery scores, was 0.131 (*p* < 0.005, Table 2) for dataset 1 and 0.365 (*p* < 0.05, Table 2) for dataset 2.

The correlation coefficients obtained with a no-feature selection procedure, between the predicted JOA recovery rate and the actual JOA recovery rate, were 0.35 (*p* = 0.01) for dataset 1 and 0.35 (*p* = 0.03) for dataset 2. The correlation coefficients obtained with a feature selection procedure, between the JOA recovery rate and the actual JOA recovery rate, were 0.62 (35 FC pairs) (*p* = 0.004) for dataset 1 and 0.52 (27 FC pairs) (*p* = 0.002) for dataset 2. The RMSE obtained with a no-feature selection procedure, between the predicted JOA recovery rate and the actual JOA recovery rate, was 1.831 (*p* < 0.05, Table 2) for dataset 1 and 4.752 (*p* < 0.05, Table 2) for dataset 2. The RMSE obtained with a feature selection procedure, between the JOA recovery rate and the actual JOA recovery rate, was 1.549 (*p* < 0.005, Table 2) for dataset 1 and 4.342 (*p* < 0.005, Table 2) for dataset 2.

**Figure 6:**
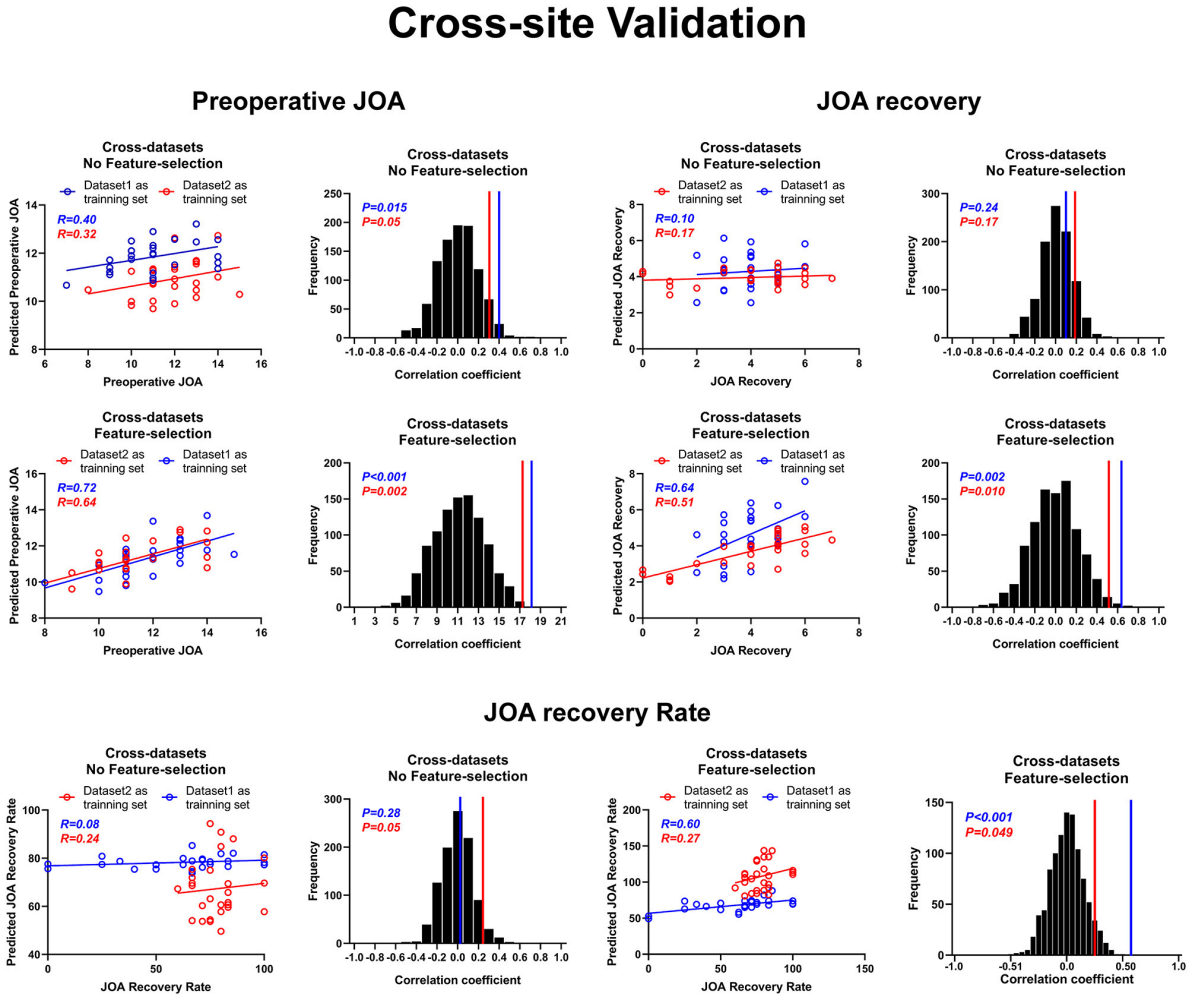
Cross-site validation for prediction analyses. Cross-site validation of the prediction of both preoperative JOA scores and JOA recovery. JOA, Japanese Orthopedic Association. JOA recovery = postoperative JOA scores minus preoperative JOA scores.

Figure 6 presents the results for cross-site validation. The correlation coefficients between the predicted preoperative JOA scores and the actual preoperative JOA scores for training sets were 0.40 (*p* = 0.01) for dataset 1 and 0.32 (*p* = 0.05) for dataset 2, respectively. The RMSE between the predicted preoperative JOA scores and the actual preoperative JOA scores for training sets was 0.347 (*p* > 0.05, Table 2) for dataset 1 and 0.326 (*p* > 0.05, Table 2) for dataset 2. After feature selection, the correlation coefficients of the training sets were 0.72 (42 FC pairs) (*p* < 0.001) for dataset 1 and 0.64 (*p* = 0.002) (37 FC pairs) for dataset 2. The RMSE of the training sets was 0.326 (*p* < 0.001, Table 2) for dataset 1 and 0.236 (*p* < 0.005, Table 2) for dataset 2.

The correlation coefficients between the predicted JOA recovery scores and the actual JOA recovery scores for the training sets were 0.10 (*p* = 0.24) for dataset 1 and 0.17 (*p* = 0.17) for dataset 2. The RMSE between the predicted JOA recovery scores and the actual JOA recovery scores for the training sets was 0.363 (*p* > 0.05) for dataset 1 and 0.263 (*p* > 0.05) for dataset 2. After feature selection, the correlation coefficients for the training sets were 0.64 (*p* = 0.002) for dataset 1 (31 FC pairs) and 0.51 (*p* = 0.01) for dataset 2 (26 FC pairs). After feature selection, the RMSE for the training sets was 0.260 (*p* < 0.005) for dataset 1 and 0.258 (*p* < 0.05) for dataset 2.

The correlation coefficients between the predicted JOA recovery rate and the actual JOA recovery rate for the training sets were 0.08 (*p* = 0.28) for dataset 1 and 0.24 (*p* = 0.05) for dataset 2. The RMSE between the predicted JOA recovery rate and the actual JOA recovery rate for the training sets was 6.736 (*p* > 0.05, Table 2) for dataset 1 and 5.860 (*p* > 0.05, Table 2) for dataset 2. After feature selection, the correlation coefficients for the training sets were 0.60 (*p* = 0.001) for dataset 1 (33 FC pairs) and 0.27 (*p* = 0.049) for dataset 2 (15 FC pairs). The RMSE for the training sets was 3.560 (*p* < 0.001, Table 2) for dataset 1 and 4.475 (*p* < 0.05, Table 2) for dataset 2. Further, Bland–Altman analyses revealed that 95% of points of all prediction analyses were within the LOA (see Supplementary Materials, Supplementary Figures 1–3).

The actual LOA of JOA prediction for dataset 1 was from −2.67 to 2.71 and was from −1.76 to 1.93 after feature selection. The LOA of JOA recovery prediction for dataset 1 was from −1.97 to 2.02 and was from 1.32 to 1.39 after feature selection. The LOA of JOA recovery rate prediction for dataset 1 was from −18.44 to 19.51 and was from −16.62 to 15.46 after feature selection. The LOA of JOA prediction for dataset 2 was from −2.71 to 2.74 and was from −2.83 to 2.71 after feature selection. The LOA of JOA recovery prediction for dataset 2 was from −3.73 to 3.65 and was from −3.65 to 3.93 after feature selection. The LOA of JOA recovery rate prediction for dataset 2 was from −50.16 to 50.43 and was from −45.52 to 46.22 after feature selection.

For cross-site validation, the LOA of JOA prediction for dataset 1 as training set was from −2.40 to 4.02 and was from −3.85 to 2.17. The LOA of JOA recovery prediction for dataset 1 as training set was from −2.91 to 2.36 and was from −3.77 to 3.79 after feature selection. The LOA of JOA recovery rate prediction for dataset 1 as training set was from −40.74 to 18.86 and was from −7.23 to 66.70 after feature selection. The LOA of JOA prediction for dataset 2 as training set was from −3.83 to 1.97 and was from −2.79 to 1.75. The LOA of JOA recovery prediction for dataset 2 as training set was from −1.70 to 3.04 and was from −2.37 to 3.02 after feature selection. The LOA of JOA recovery rate prediction for dataset 2 as training set was from −36.48 to 69.07 and was from −39.63 to 51.88 after feature selection.

## Discussion

In this study, we conducted MVPAs of FC in patients with CSM, including (1) univariate analyses for revealing the differences for FC between CSM patients and HCs; (2) classification between CSM patients and HCs; (3) prediction of preoperative JOA scores; and (4) prediction of JOA recovery rate and JOA recovery scores. Our results demonstrated that rs-FC combined with SVM could successfully classify CSM patients from HCs and that rs-FC combined with SVR could successfully predict the neurological recovery in CSM patients. These results further indicated that MVPA approach could capture the rs-FC pattern abnormalities in CSM patients and could be used as a potential biomarker for predicting the surgical outcomes in CSM patients.

CSM is commonly seen in practice, and the preoperative grading of CSM severity and prognosis prediction are matters of great concern for clinical surgeons. Conventional cervical MRI (i.e., T1 and T2) has been used to diagnose CSM for the past decades; however, its utility for predicting CSM prognosis has been controversial (36). Several metrics measuring the morphologic changes of the spinal cord has been shown to be not so reliable for predicting surgical outcomes (37). To resolve this issue, several neuroimaging approaches, including diffusion tensor imaging (DTI) (38–40), proton magnetic resonance spectroscopy (41, 42), and electromyography combined with conventional MRI (43), have been proposed for prognostic use in CSM. It has been shown that the DTI analysis of spinal tracts might provide additional information for prognosis of CSM (39, 40). Moreover, it has been also shown that the metabolic changes of the sensorimotor cortices were also associated with the neurological recovery following decompression surgery (41, 42, 44). Other approaches, such as electromyography, have also been shown to provide prognostic information for CSM (43). However, these techniques were not easily accessible in clinical practices (i.e., long acquisition time and being invasive). Therefore, the need for simple, accurate, and non-invasive imaging biomarkers for prognostic use in CSM patients is warranted.

In recent years, researchers turned their attention to brain rs-fMRI, which is easily acquired and non-invasive in clinical practice. At the first glance, it seems surprising to develop a prognostic biomarker based on brain rs-fMRI given that CSM is not a primary cortical disorder. However, previous studies have shown that the resting-state and task fMRI were useful for developing potential neural biomarkers for assessing preoperative sensorimotor deficits in CSM patients (11). A seed-based FC study conducted by Peng et al. showed that the FCs between the anterior and the cerebellum, the anterior thalamus, and the cuneus significantly increased and positively correlated with preoperative JOA scores. Furthermore, Zhou et al. and Peng et al. observed that increased FCs between the anterior thalamus and precentral gyrus positively correlated with the upper limb motor function in CSM patients. Moreover, the resting-state FC between the thalamus and the pre/postcentral gyrus was correlated with the severity of long-term spinal cord injury (12, 45).

Recently, Takenaka and Kan (11) reported that the FC between the visual cortex and the frontal gyrus is associated with the 10-s test results and could predict postoperative neurological recovery in CSM patients. Besides, in our previous study, we demonstrated a significant correlation between the increased FC and preoperative JOA scores (46). Despite these studies demonstrating that several rs-fMRI metrics may be useful for presurgical evaluation in CSM patients, these studies only conducted univariate correlation analysis for revealing the linear association between brain metrics and outcome measures. Therefore, the pattern (i.e., consisted of multi-voxels or multi-connections) information, which could be detected by the MVPA, may be ignored by conventional approach.

In this study, we conducted an FC analysis and constructed the whole-brain network. We tested the utility of classification of CSM patients from HCs using FCs as features *via* an SVM. We obtained good performance both within datasets and across two independent datasets. Moreover, the model's performances were also increased after feature selection. Our findings indicated that the classification accuracies were high within each dataset and could be generalized between two independent datasets acquired by different MR machines. Therefore, our findings suggest that the rs-FC may be instrumental in the diagnosis of CSM in patients.

Moreover, we assessed the potential utility of rs-FC in the presurgical evaluation of CSM using the SVR. We obtained successful regression between rs-FC and the preoperative neurological function (e.g., preoperative JOA scores) in CSM patients since all correlation coefficients were above 0.4 before feature selection and above 0.6 after feature selection (all *p*-values < 0.05 after permutation test and family-wise error (FWE) correction). These findings also showed good generalization across the two datasets. Therefore, our current results provided preliminary evidence that the pattern of rs-FC is associated with the presurgical neurological function in CSM patients and may aid the evaluation of CSM patients for research purposes. It is obvious that there are differences in the predicted and actual JOA scores. Two main reasons may contribute to these differences. First, rs-fMRI data constitute multiple sources of noise during data collection (e.g., respiratory or cardiac noise). Despite that the preprocessing steps could largely increase the noise-to-signal ratio of rs-fMRI data. There still were unexpected noises, which may affect the accuracy of the prediction analysis. Second, although JOA scale is the most commonly used and robust clinical measure for evaluating the severity of CSM, it only measures the sensorimotor aspect of the CSM patients (e.g., sensory, motor, and bowel and bladder deficits). Other psychological factors (e.g., cognitive deficits and depression), which have also shown to be associated with the CSM, could not be evaluated by the JOA scale. Therefore, such measurement error may also contribute to the prediction error between actual JOA scores and predicted JOA scores.

Besides, we explored the association between the rs-FC and prognosis of CSM *via* SVR, using preoperative rs-FCs as features and the sensorimotor recovery following spinal cord decompression surgery (JOA recovery or JOA recovery rate) as labels. Despite the successful prediction of the JOA recovery scores in each dataset, the correlation coefficients were relatively low except for the prediction of JOA scores after feature selection in dataset 1. The cross-site prediction performances were also relatively poor (R = 0.10/0.17 before feature selection; R = 0.51/0.64 after feature selection) compared with the prediction of preoperative JOA scores. This may be attributable to various factors such as age, disease duration, presurgical neurological state, spinal cord DTI signal, and surgical approaches, which affect the prognosis of CSM (2, 4, 47, 48); thus, it would make the prediction harder than we expected. It is worth mentioning that the outcome of the JOA recovery prediction by SVR is generally poor. Interestingly, within the low performance, the model appears to perform better on dataset 1 than on dataset 2, though the opposite was true in classifying patients from HCs. This is likely to reflect the fact that myriad factors in postoperative recovery may not be captured by the rs-FC data. Moreover, the non-generalizability of the cross-site prediction before feature selection may be due to the different sets of features selected during the training process, thus making the cross-site prediction harder. It is also worth mentioning that the poor prediction for JOA recovery could also be attributed to the SVR itself. Before feature selection, there were 6,670 features included in the SVR model; however, there were only <100 samples for training the model. Overfitting of these models could also be a major cause of poor prediction. The improvements of prediction accuracy after feature selection could further support this speculation due to the fact that feature selection procedure could remove redundant features to some extent.

For clinical significance, the Bland–Altman analyses were performed to reveal the clinical significance of the prediction analyses. In the case for JOA prediction, the minimum clinically important difference of the JOA has been shown to be 1–2 points (49); and the minimum clinically important difference of the JOA recovery rate has shown to be 52.8% in CSM patients (50). In our current analysis, the 95% LOA exceeded these, meaning that the predicted data could deviate from the actual JOA score (or actual JOA recovery rate) by more than what is accepted as a clinically meaningful change. These results indicated that predicting CSM-related outcomes is not yet robust enough for accurate predictions (e.g., for clinical purpose), though it does show promise and could be developed with a bigger dataset or with other outcome variables.

## Limitations

Since our study only used rs-FC as features to classify CSM patients and predict clinical measures, other rs-fMRI metrics and feature fusion approach are needed in the future to develop more accurate diagnostic and prognostic models for CSM. Moreover, our current study is a retrospective study and lack repeatability analysis (when tested on the same individual at two different time points under the same conditions). Therefore, it may be a potential confounder of unknown significance. As mentioned above, our study is a retrospective study; therefore, the sample size and the statistical power have not been estimated, and the prediction analyses were performed after the data collection of follow-up information. Prospective study using more rigorous statistical analyses and directly comparing the prediction accuracy between orthopedic surgeon and machine learning techniques is required in the future. Furthermore, we did not collect postoperative fMRI data due to possible artifacts and MRI heating of implants. Therefore, we recommend long-term follow-up before postoperative data collection for safety. Additionally, spinal cord MR data, including the DTI, diffusion spectrum imaging (DSI), and functional scan, should be collected in the future to obtain more information on CSM. Future studies may need to add the clinical information and spinal cord MR data to the prediction model to improve the prediction performance.

## Data Availability Statement

The raw data supporting the conclusions of this article will be made available by the authors, without undue reservation.

## Ethics Statement

The studies involving human participants were reviewed and approved by Tianjin Medical University General Hospital. The patients/participants provided their written informed consent to participate in this study.

## Author Contributions

QS and FY designed this study. SW, RZ, and HT collected the data. RZ wrote the manuscript. XG revised the manuscript. All authors contributed to the article and approved the submitted version.

## Funding

This study was funded by the Tianjin Medical University Cancer Institute and Hospital Fund (Grant No. B19 02).

## Conflict of Interest

The authors declare that the research was conducted in the absence of any commercial or financial relationships that could be construed as a potential conflict of interest.

## Publisher's Note

All claims expressed in this article are solely those of the authors and do not necessarily represent those of their affiliated organizations, or those of the publisher, the editors and the reviewers. Any product that may be evaluated in this article, or claim that may be made by its manufacturer, is not guaranteed or endorsed by the publisher.
