# Supplementary material for: Identification and Therapeutic Outcome Prediction of Cervical Spondylotic Myelopathy Based on the Functional Connectivity From Resting-State Functional MRI Data: A Preliminary Machine Learning Study
Source: Front Neurol. 2021 Oct 8;12:711880. doi: 10.3389/fneur.2021.711880 (PMC8531403; doi:10.3389/fneur.2021.711880)
Supplement: Supplementary file 1 [file Data_Sheet_1.docx]

**Supplementary materials**

**Surgical procedures**

The patients included in our current study had different responsible levels, however, these patients all fitted the surgical indications for anterior cervical discectomy and fusion (ACDF). Therefore, the patients we include were all underwent the ACDF surgery using a Smith-Robinson method. The detailed procedures were as follows: All patients received ACDF by the same surgical team. A standard anterolateral approach from the right side was performed to expose the targeted segment. After distraction screws were placed into the adjacent vertebral bodies, the Caspar distractor was used to open the intervertebral space. The compressive materials including herniated disc and posterior longitudinal ligament were removed. The cartilage endplate was also removed, and the bony endplate was retained to prevent subsidence. Then, an appropriate-sized cage (Medtronic Sofamor Danek, Memphis, TN, USA) filled with allograft bone was used. The size of the cage was determined by intraoperative evaluation using a trial cage to achieve the initial stability. After implantation of the cage, the Caspar distractor was released and the stability of the cage was confirmed. Finally, the anterior plate system was applied (Medtronic Sofamor Danek, Memphis, TN, USA). Postoperatively, all patients were encouraged to resume their normal activities as soon as possible with a cervical collar to avoid overextension for 4 weeks.

**Supplementary Figures**

**S-Figure 1**

**
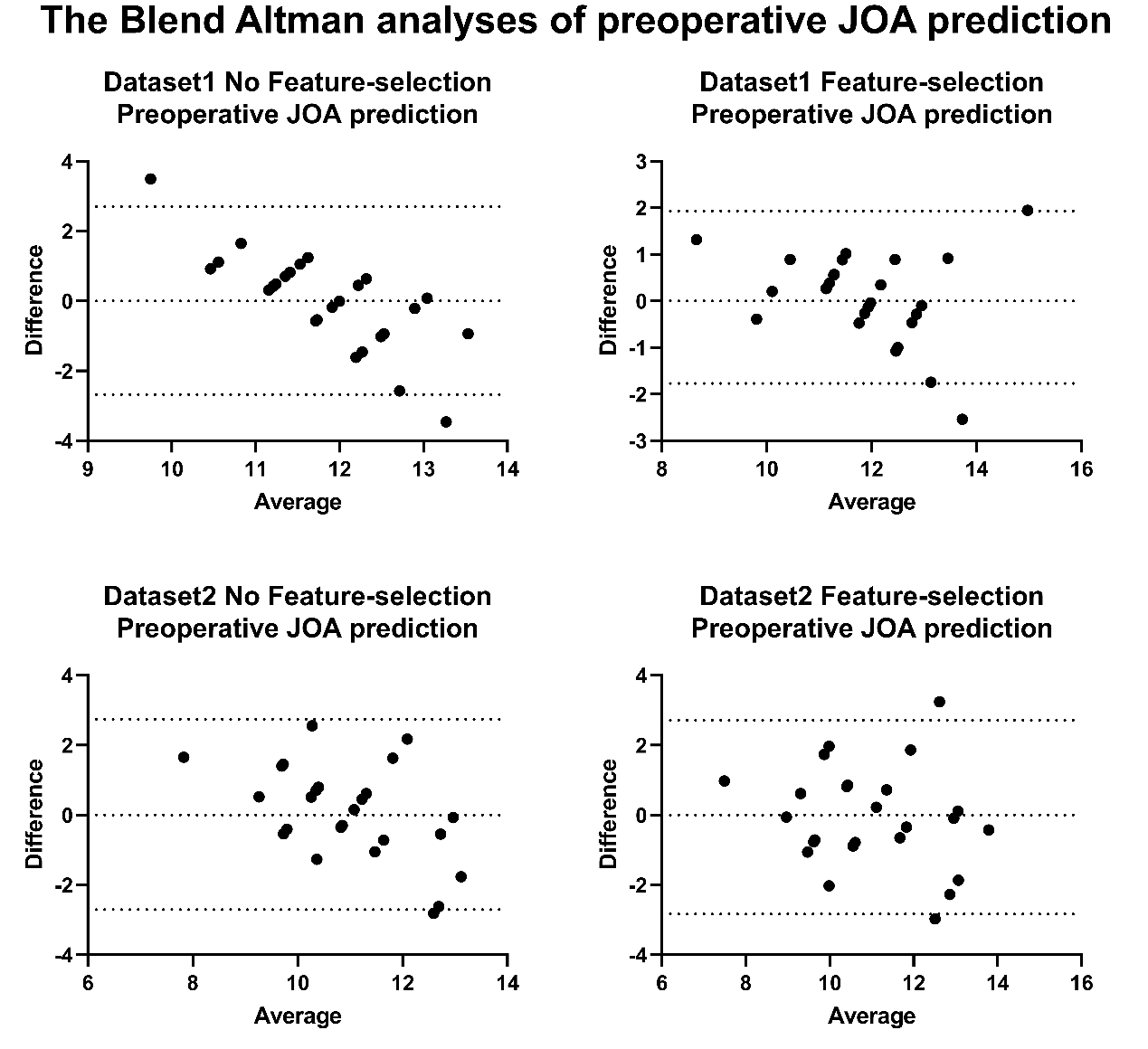
**

The results of Bland-Altman analyses for within-dataset preoperative JOA prediction.

**S-Figure 2**

**
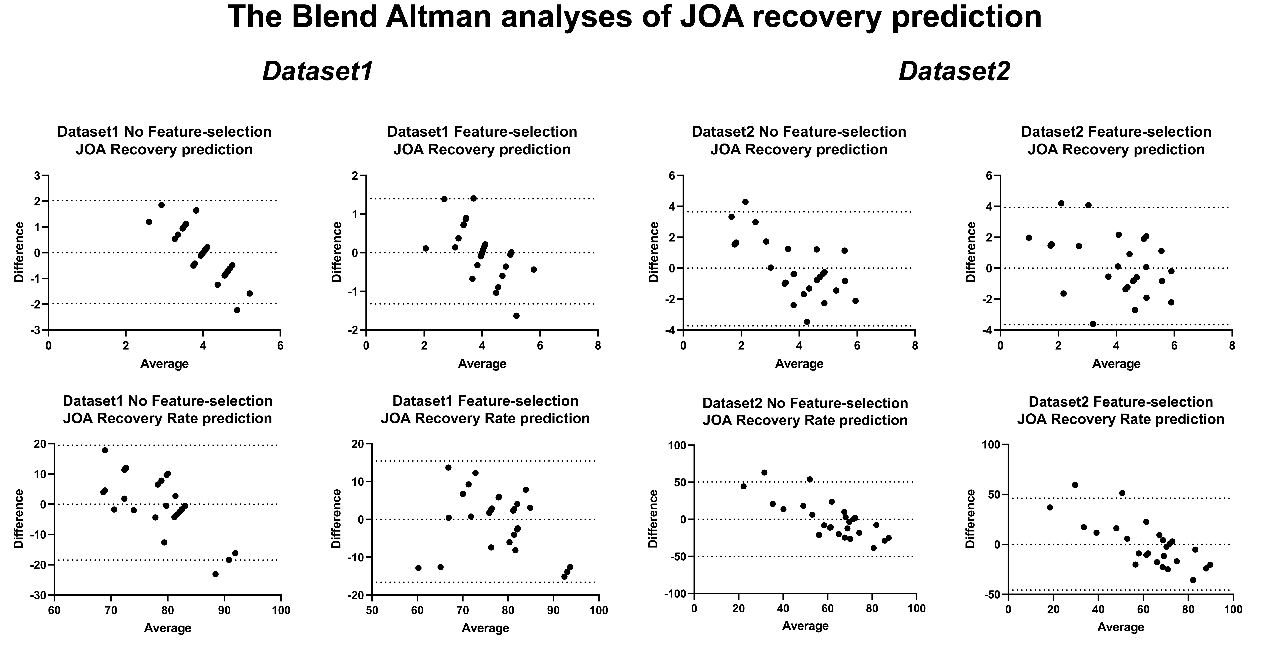
**

The results of Bland-Altman analyses for within-dataset JOA recovery prediction.

**S-Figure 3**

**
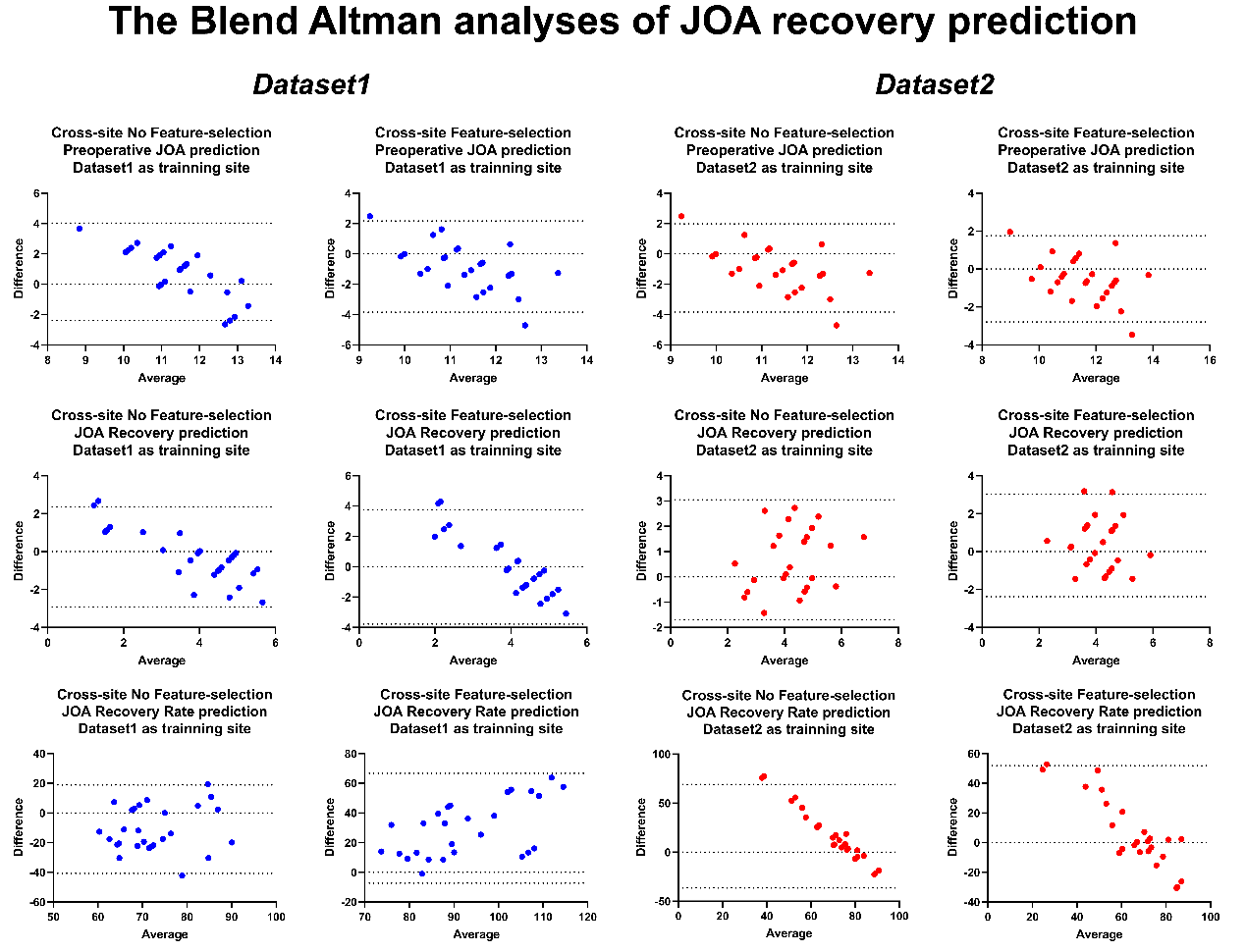
**

The results of Bland-Altman analyses for cross-site validation prediction analyses.
